# Insights into the nutritional properties and microbiome diversity in sweet and sour yogurt manufactured in Bangladesh

**DOI:** 10.1038/s41598-021-01852-9

**Published:** 2021-11-22

**Authors:** S. M. Rafiqul Islam, Afsana Yeasmin Tanzina, Md Javed Foysal, M. Nazmul Hoque, Meheadi Hasan Rumi, A. M. A. M. Zonaed Siddiki, Alfred Chin-Yen Tay, M. Jakir Hossain, Muhammad Abu Bakar, Mohammad Mostafa, Adnan Mannan

**Affiliations:** 1 Department of Genetic Engineering and Biotechnology, Faculty of Biological Sciences, University of Chittagong, Chattogram, 4331 Bangladesh; 2 School of Molecular and Life Sciences, Curtin University, Bentley, WA 6102 Australia; 3 Department of Genetic Engineering and Biotechnology, Shahjalal University of Science and Technology, Sylhet, 3114 Bangladesh; 4 Department of Gynecology, Obstetrics and Reproductive Health, Bangabandhu Sheikh Mujibur Rahman Agricultural University, Gazipur, 1706 Bangladesh; 5 Department of Pathology and Parasitology, Chattogram Veterinary and Animal Sciences University, Chattogram, 4225 Bangladesh; 6 Helicobacter Research Laboratory, The Marshall Centre, University of Western Australia, Perth, WA 6009 Australia; 7 Forest Chemistry Division, Bangladesh Forest Research Institute, Chattogram, 4211 Bangladesh; 8 Bangladesh Council of Scientific and Industrial Research (BCSIR) Laboratories, Chattogram, 4220 Bangladesh

**Keywords:** Biochemistry, Biological techniques, Biotechnology, Microbiology, Molecular biology

## Abstract

Yogurt is one of the most frequently consumed dairy products for nutritional benefits. Although yogurt is enriched with probiotics, it is susceptible to spoilage because of the presence of pathogenic microbes. Spoiled yogurt if consumed can cause food-borne diseases. This study aimed to assess the nutritional composition and microbiome diversity in yogurt manufactured in Bangladesh. Microbial diversity was analyzed through high-throughput sequencing of bacterial 16S rRNA gene and fungal internal transcribed spacer (ITS) region. From nutritional analysis, significantly (*P* < 0.05) higher pH, fat, moisture, total solid and solid-non-fat contents (%) were observed in sweet yogurt. Following the classification of Illumina sequences, 84.86% and 72.14% of reads were assigned to bacterial and fungal genera, respectively, with significantly higher taxonomic richness in sour yogurt prepared from buffalo. A significant difference in bacterial (*P*_permanova_ = 0.001) and fungal (*P*_permanova_ = 0.013) diversity between sweet and sour yogurt was recorded. A total of 76 bacterial and 70 fungal genera were detected across these samples which were mostly represented by Firmicutes (92.89%) and Ascomycota (98%) phyla, respectively. This is the first study that accentuates nutritional profiles and microbiome diversity of Bangladeshi yogurt which are crucial in determining both active and passive health effects of yogurt consumption in individuals.

## Introduction

Fermented dairy products have received increasing attention due to their enhanced nutritional and sensory properties, micro- and macronutrients, and extended shelf-life^1,2^. These products are associated with numerous health benefits through providing the consumer with both readily metabolizable nutrients and beneficial microorganisms^3,4^. Globally more than 400 fermented foods are manufactured from milk, among which yogurt, also known as “Dahi” in Indian subcontinent, is the most popular for its sensory attributes. As people became conscious about the nutritional and health benefits of yogurt, its consumption started increasing gradually^5,6^. Yogurt is now being manufactured in amounts and varieties with different fat contents, flavors and tastes. Sweet and sour yogurt are the two basic forms of yogurt different in taste and manufacturing process, therefore, contain a plethora of diverse microorganisms. Sour yogurt is prepared from the fermentation of lactose in milk by lactic acid bacteria (LAB), which produce lactic acid that act on milk protein to give yogurt sour taste^1,7^. Conversely, when various flavors and sweetening agents are added to the yogurt, it is called sweet yogurt. Sweet yogurt is made of curd formed with rennet from cow milk set sweet and cooked rapidly to a very firm consistency^5,7,8^. This dairy product is a good source of protein, vitamins (e.g., vitamin A, vitamin B2, vitamin B5 and vitamin B12), minerals (including sodium, potassium, calcium, magnesium, iron, zinc and copper) and some key fatty acids (e.g., linoleic acid, palmitic acid and myristic acid)^9,10^. Moreover, yogurt is used as a major source of probiotics, which are beneficial bacteria thought to improve human health^5^. The beneficial effects of yogurt depend on both qualitative and quantitative composition of the constituent microflora in the yogurt, which need to be ascertained. Yogurt is vulnerable to spoilage by pathogenic microorganisms. Compromise and imbalance in nutritional properties and poor product quality also reduce the shelf-life of yogurt, causing food waste. Often, consuming substandard quality yogurt can lead to food-borne diseases (e.g., diarrhea and gastrointestinal infection). In addition, the quality of yogurt is an important variable because of the influence of different factors such as optimal incubation time and temperature, uninterrupted fermentation cycle, firmness or durability of raw ingredients as well as final product etc.^2^ Hence, there is an urgent need to establish an efficient method to explore the nutritional properties and microbiota diversity in yogurt manufactured in Bangladesh for the improvement of its food value and safety.

The content of trace elements in dairy milk and products has begun to be more widely studied, particularly in industrialized and polluted regions, since it is considered as a good bioindicator of pollution of the agricultural environment^11,12^. According to FAO (Food and Agriculture Organization), regular yogurt contains 5–6% protein, 8.25% solid-not-fat (SNF), and 3.5% fat, though the fat content varies from 0 to 3.5% based on the type of yogurt^9^. In Bangladesh, yogurt is traditionally manufactured by fermentation of cow and buffalo milk or a mixture of them at an optimal pH 4.0–4.6 primarily through the action of *Streptococcus salivarius* subsp. *thermophilus*, and *Lactobacillus delbrueckii* subsp. *bulgaricus*^8^. Yogurt, prepared from cow and buffalo milk, is a rich source of diverse microbial communities, which vary within different categories of yogurt (sweet and sour) and different manufacturers. The microbiome diversity and composition of the yogurt can thereby regulate the development of the organoleptic properties of yogurt, nutrient composition, shelf-life and associated safety^13^.

Though the conventional starter culture of yogurt includes only *S. thermophilus* and *L. bulgaricus*, its characteristic pH favors the growth of other organisms (e.g., *Lactococcus lactis* subsp. *diacetylactis*, *Lactococcus cremoris* and *Lactobacillus rhamnosus*) which contribute to yogurt viscosity, appealing aroma and taste, and thought to have probiotic effects^5^. The major drawback of these conventional culture-based screening methods is their inability to reveal microbes that are insensitive to culture but responsible for a greater impact on fermented foods. Recent advances in culture-independent techniques such as high-throughput sequencing and next generation sequencing technology along with advanced bioinformatics and computational tools during the last decade have revolutionized the research on food microbial ecology, leading to consider microbial populations as consortia^14–16^. By employing this technology, unculturable microbial flora including both beneficial and pathogenic bacteria, as well as fungi have been successfully identified in various naturally fermented milk products^16,17^. One of the recent studies conducted using both culture-dependent and culture-independent methods reported *Lactobacillus* and *Streptococcus* as dominant bacteria and *Kodamaea*, *Clavispora*, *Candida*, and *Tricosporon* as dominant fungal genera in four traditional Bangladeshi fermented milk products (dahi, chanar-misti, paneer, and borhani)^18^. Another study conducted by Rashid et al. identified bacterial species including *L. bulgaricus*, *L lactis*, *L. fermentum*, *S. thermophilus*, *S. bovis*, *E. faecium*, *L. mesenteroides*, *L. dextranicum*, *Lc. lactis*, *Lc. raffinolactis*, *P. pentosaceus* in traditional fermented milk Dahi from different parts of Bangladesh^19^. However, these studies did not indicate any characteristic microbial features of a particular brand or taste type.

Most of the previous studies focused on yogurt included biochemical metabolite analysis, flavor identificationt, quantification, and sensory characterization that drive the consumer likings of yogurt^20–22^. Given the fact that the microbial community of yogurt highly influences the taste characteristics of yogurt, recent trends in yogurt-related research have focused more on technologies improving the yogurt qualities by adding functional ingredients^20,23–25^. However, each study focused only on one aspect of yogurt, rather than considering two or more factors jointly in one research work. To our knowledge, no comprehensive research has been carried out in Bangladesh as of now for investigating the presence of nutritional elements and trace minerals in yogurt. Besides, detailed knowledge on biochemical potential and variations in microbiome composition along with diversity among different brands and taste types is yet to be analyzed. To manufacture high-quality and nutritious yogurt, it is important to conduct a study with a view to detecting microbial flora including beneficial and pathogenic bacteria and fungi.

**Figure 1:**
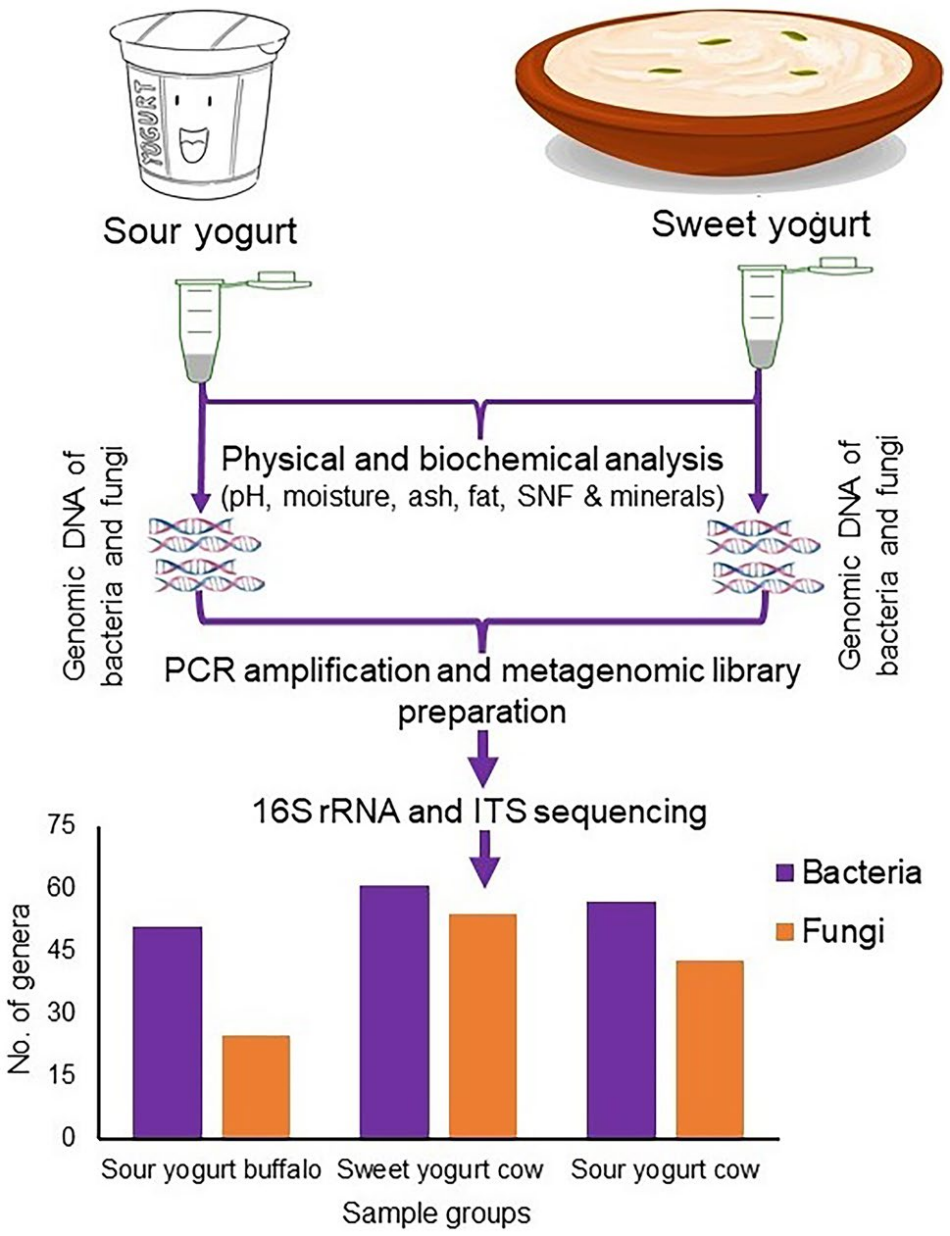
Schematic representation of nutritional and microbiome study in sweet and sour yogurt samples produced from cow and buffalo milk manufactured by seven reputed brands in Bangladesh.

This study was undertaken to assess the nutritional quality and detect microbiome diversity of sweet and sour yogurt samples produced from cow and buffalo milk manufactured by seven reputed brands in Bangladesh (Fig. 1). This work was conducted in two stages; in stage one, the biochemical parameters and trace minerals of the yogurt samples were determined to ensure their nutritional status and safety and in stage two, the microbial (bacteria and fungi) community was scrutinized through targeted sequencing of the 16S rRNA and ITS genes under Illumina platform for uncovering the microbial consortia and their activities in yogurt. The obtained results will allow the regulatory bodies to scale the nutritional profile of yogurt manufactured in Bangladesh as well as give them an overview on the microbial community present in each sample. As both the nutritional elements and microbiome in yogurt contribute to its taste, quality and effects on human health, the outcomes of this study will aid regulatory authorities in monitoring the quality and microbiota composition of yogurt, and maintain proper guidelines for standard yogurt manufacturing in Bangladesh.

## Results

### Nutritional composition of yogurt

#### Analysis of biochemical parameters

The quality and safety of yogurt depends on its nutritional composition and microbiome diversity as well as quality of the starter culture. To assess yogurt quality, the nutritional properties of yogurt in terms of biochemical parameters and trace minerals were examined in this study. Biochemical parameters of yogurt samples including pH, fat, moisture, total solid (TS), solid-not-fat (SNF) and ash contents are summarized in Table 1. During the commercial yogurt manufacturing process, pH needs to be maintained in order to prevent the generation of rancid smell, taste and flavor. A high pH indicates improper fermentation and allows many undesirable microorganisms to grow in the medium. We found significant differences (*P* < 0.05) in pH values of all yogurt varieties studied in this report. The mean pH values of the yogurt samples remained slightly acidic and, on an average, ranged from 5.28 to 6.33. The Sweet Brand 6 (SwV5) had the highest average pH value of 6.33 ± 0.13 whereas the Sour Brand 7 (SoV5) showed the lowest pH (5.28 ± 0.25) (Table 1). Interestingly, no significant difference was found for fat content of the analyzed yogurt samples. The average fat content of the samples varied from 0.25 to 2.52% (*w/w*). The Sweet Brand 1 (SwV1) had the highest fat content (2.52 ± 3.68%) while the lowest fat content (0.25 ± 0.05%) was recorded in Sour Brand 3 (SoV2) (Table 1).

**Table 1:** Biochemical parameters of Bangladeshi yogurt of different brands and tastes.

Brand	Variety	Parameters (%, *w/w*; except pH)					
		pH	Fat^A^	Moisture	TS	SNF	Ash^A^
Sweet Brand 1	SwV1	5.8^ab^ ± 0.16	2.52 ± 3.68	82.1^a^ ± 1.16	17.90^a^ ± 1.16	15.37^a^ ± 3.36	0.86 ± 0.23
Sweet Brand 2	SwV2	6.2^a^ ± 0.04	0.93 ± 0.30	78.88^b^ ± 0.38	21.11^b^ ± 0.38	20.18^b^ ± 0.58	0.93 ± 0.30
Sweet Brand 3	SwV3	6.32^a^ ± 0.17	0.9 ± 0.62	80.53^ab^ ± 0.84	19.47^ab^ ± 0.84	18.57^ab^ ± 1.13	1.13 ± 0.31
Sweet Brand 4	SwV4	6.28^a^ ± 0.01	0.8 ± 0.2	60.72 ± 0.80	39.29 ± 0.80	38.48 ± 0.74	0.80 ± 0.20
Sweet Brand 6	SwV5	6.33^a^ ± 0.13	2.32 ± 1.80	75.83 ± 0.73	24.17 ± 0.73	21.85^bc^ ± 1.07	0.80 ± 0.20
Sour Brand 2	SoV1	5.84^ab^ ± 0.11	1.41 ± 0.66	82.44^a^ ± 0.69	17.56^a^ ± 0.69	16.16^a^ ± 0.80	1.06 ± 0.30
Sour Brand 3	SoV2	5.71^ab^ ± 0.09	0.25 ± 0.05	87.59 ± 0.53	12.41 ± 0.53	12.16^a^ ± 0.51	1.06 ± 0.31
Sour Brand 4	SoV3	5.98^a^ ± 0.03	2.15 ± 1.91	79.17^b^ ± 0.49	20.82^b^ ± 0.49	18.67^ab^ ± 1.46	1.26 ± 0.31
Sour Brand 5	SoV4B	6.28^a^ ± 0.01	1.6 ± 0.2	84.74 ± 0.64	15.26 ± 0.64	13.66^a^ ± 0.53	1.13 ± 0.42
Sour Brand 7	SoV5	5.28 ± 0.25	2.19 ± 0.19	72.06 ± 0.82	27.94 ± 0.82	25.75^c^ ± 1.01	1.0 ± 0.20
Level of significance		***	NS	***	***	***	NS

TS = Total solid, SNF = Solid-not-fat, SwV = Sweet variety, SoV = Sour variety, Mean values with different superscripts (^a, b, c, ab, bc^) in the same column differed significantly while those left with no superscripts differed with all, A = No mean value showed any significant difference among each other hence were left single i.e. without any superscripts, *** = Significant at 5% level (*P* < 0.05), NS = Non-significant, The values of all parameters were recorded as mean ± SD.

Moisture content (MC) is one of the most common properties of food products due to a number of reasons like food quality, microbial stability along with legal and labeling requirements. The MC of the yogurt samples were in the range of 60.72 ± 0.80 to 87.59 ± 0.53% and varied significantly (*P* < 0.05) across the sample groups (Table 1). The average MC was much higher for all sweet Brands compared to sour Brands except Sweet Brand 4. The Sour Brand 3 had the highest MC (87.59 ± 0.53%) and lowest MC (60.72 ± 0.80%) was recorded in Sweet Brand 4 (SwV4). Likewise, the TS and SNF values differed significantly (*P* < 0.05) across the sample groups. We found the highest amount of TS (39.29 ± 0.80%) and SNF (38.48 ± 0.74%) in Sweet Brand 4 yogurt which remained lowest (TS; 12.41 ± 0.53% and SNF; 12.16 ± 0.51%) in Sour Brand 3 yogurt (Table 1). However, after removing the organic residues present in samples, Sweet Brand 4 and Sweet Brand 6 yogurt samples yielded the lowest ash content (0.80 ± 0.20%), while the highest amount of ash content (1.26 ± 0.31%) was found in Sour Brand 4 (SoV3) yogurt. No significant difference in ash content was found among the sample varieties.

#### Analysis of mineral contents

Yogurt is an important source of essential minerals. The contents of minerals including Na, K, Ca, Mg, Fe, Zn, and Cu in yogurt samples of different brands and tastes are given in Table 2. The mean value for Na content ranged from 593.50 ± 65.55 to 1052.65 ± 332.42 mg/kg indicating its highest and lowest content in Sour Brand 4 and Sour Brand 3, respectively. The Brand 4 of sour yogurt had the highest content of K (2744.54 ± 669.79 mg/kg), Ca (2442.27 ± 92.21 mg/kg), and Mg (272.42 ± 27 mg/kg). The lowest concentration of K (719.36 ± 135.50 mg/kg) was found in Sour Brand 5 (SoV4B), while the Sweet Brand 4 had the least mean content of Ca (1115.40 ± 354.57 mg/kg) and Mg (107.72 ± 20.05 mg/kg). Among these trace elements, the value of K varied within a broad range of 719.36 ± 135.5–2744.52 ± 669.79 mg/kg.

**Table 2:** The mineral contents in Bangladeshi yogurt of different brands and tastes (sweet and sour).

Brand	Variety	Mineral contents (mg/kg)						
		Sodium^A^	Potassium	Calcium	Magnesium	Iron	Zinc	Copper^A^
Sweet Brand 1	SwV1	860.39 ± 311.19	1755.96^a^ ± 386.41	2043.44^ac^ ± 456.92	176.52^a^ ± 20.27	16.36 ± 0.72	7.78^a^ ± 0.90	2.60 ± 0.29
Sweet Brand 2	SwV2	791.74 ± 53.83	1958.41^ab^ ± 211.74	1787.10^ab^ ± 166.03	152.93^a^ ± 31.62	9.63 ± 1.21	6.45^ab^ ± 2.01	3.97 ± 0.58
Sweet Brand 3	SwV3	780.62 ± 187.67	1867.45^a^ ± 100.17	1947.28^ac^ ± 327.73	176.29^a^ ± 44.18	20.63^c^ ± 1.74	4.07^b^ ± 0.51	2.63 ± 0.50
Sweet Brand 4	SwV4	610.86 ± 107	2074.86^ab^ ± 152.65	1115.40^b^ ± 354.57	107.72^ac^ ± 20.05	6.14^b^ ± 0.41	3.91^b^ ± 0.15	1.26 ± 0.27
Sweet Brand 6	SwV5	837.37 ± 117	2255.44^ab^ ± 50.46	1612.26^bc^ ± 171.89	162.63^a^ ± 20.50	28.84 ± 1.20	8.45^a^ ± 2.03	2.33 ± 1.18
Sour Brand 2	SoV1	810.66 ± 258.41	1906.49^a^ ± 256.30	2201.93^ac^ ± 222.73	198.77^ab^ ± 20	3.73^ab^ ± 0.38	7.17^a^ ± 0.61	4.87 ± 6.02
Sour Brand 3	SoV2	593.50 ± 65.55	1529^a^ ± 22.07	1743.37^bc^ ± 163.28	154.96^a^ ± 8.71	3.37^a^ ± 0.23	5.64^ab^ ± 0.20	1.11 ± 0.75
Sour Brand 4	SoV3	1052.65 ± 332.42	2744.54^b^ ± 669.79	2442.27^a^ ± 92.21	272.42 ± 27	2.74^a^ ± 0.23	7.29^a^ ± 0.31	4.37 ± 0.58
Sour Brand 5	SoV4B	646.90 ± 45.54	719.36 ± 135.50	1955.67^ac^ ± 283.44	156.73^a^ ± 17.65	4.21^a^ ± 0.17	6.43^ab^ ± 0.39	2.58 ± 0.19
Sour Brand 7	SoV5	837.49 ± 34.55	1579.81^a^ ± 26.13	1803.35^ab^ ± 169.06	146.65^a^ ± 9.29	21.45^c^ ± 1.23	8.47^a^ ± 0.32	1.69 ± 0.12
Level of significance		NS	***	***	***	***	***	NS

SwV = Sweet variety, SoV = Sour variety, Mean values with different superscripts (^a, b, c, ac, ab, bc^) in the same column differed significantly while those left with no superscripts differed with all, A = No mean values showed any significant difference among each other hence were left single i.e. without any superscripts, *** = Significant at 5% level (*P* < 0.05), NS = Non-significant, The values of all parameters were shown as mean ± SD.

The concentration of Fe in yogurt varied from 2.74 ± 0.23 to 8.84 ± 1.20 mg/kg. The content of Fe in different brands of yogurt was statistically significant (*P* < 0.05) since the sweet yogurt always had higher mean values of Fe content except for Sour Brand 7 in which the Fe content was detected as 21.45 ± 1.23 mg/kg (Table 2). The concentration of Zn ranged from 3.91 ± 0.15 to 8.47 ± 0.32 mg/kg. The highest accumulation of Zn (8.47 ± 0.3 mg/kg) was noted in Sour Brand 7 followed by in sample Sweet Brand 6 (8.45 ± 2.03 mg/kg), while the least concentration (3.91 ± 0.15 mg/kg) was detected in Sweet Brand 4 yogurt. The amount of Cu varied within the range of 1.11 ± 0.75 to 4.87 ± 6.02 mg/kg. The maximum value was detected in Sour Brand 2 (SoV1) with the lowest content of Cu (1.11 ± 0.75 mg/kg) in Sour Brand 3. The extreme SD value with the maximum level of Cu indicates the concentration of Cu differed significantly (*P* < 0.05) among the brands of yogurt (Table 2). These results highlighted that mineral contents vary among different commercial brands (Brand 1–Brand 7) and taste types (sweet and sour) of yogurt. Although minerals represent a small portion of yogurt, they are fundamental for human health, yogurt quality and its characteristic tastes.

### Microbiome composition and diversity

Since the microbiota composition is a key factor affecting the production and quality of fermented milk products, it is important and necessary to explore desirable microbes in order to manufacture high-quality yogurt^1–3^. Yogurt microbiomes of 30 samples (sweet = 15 and sour = 15) belonging to seven different commercial brands (Brand 1–Brand 7) were analyzed through high-throughput amplicon sequencing. The sweet yogurt samples included only cow samples (n = 15), and the sour yogurt included both cow (n = 12) and buffalo (n = 3) samples. During this study, the targeted sequencing approach generated a total of 3.10 million high quality reads (with an average of 0.104 million reads per sample), of which 1.4 and 1.7 million reads were assigned into 306 bacterial and 3144 fungal OTUs (Data S1). Among the reads, 44.86% and 55.14% reads were assigned to bacterial and fungal taxa, respectively. There is a clear difference between phylogenetic profiles and microbiota quantitation obtained using 16S rRNA (V3-V4) and ITS primers. An average good’s coverage index of 0.995 for bacteria and 0.996 for fungi indicated that sequencing depth was sufficient enough to capture most of the microbial community at different taxa levels (phylum, order, genus etc.) (Data S1).

**Figure 2:**
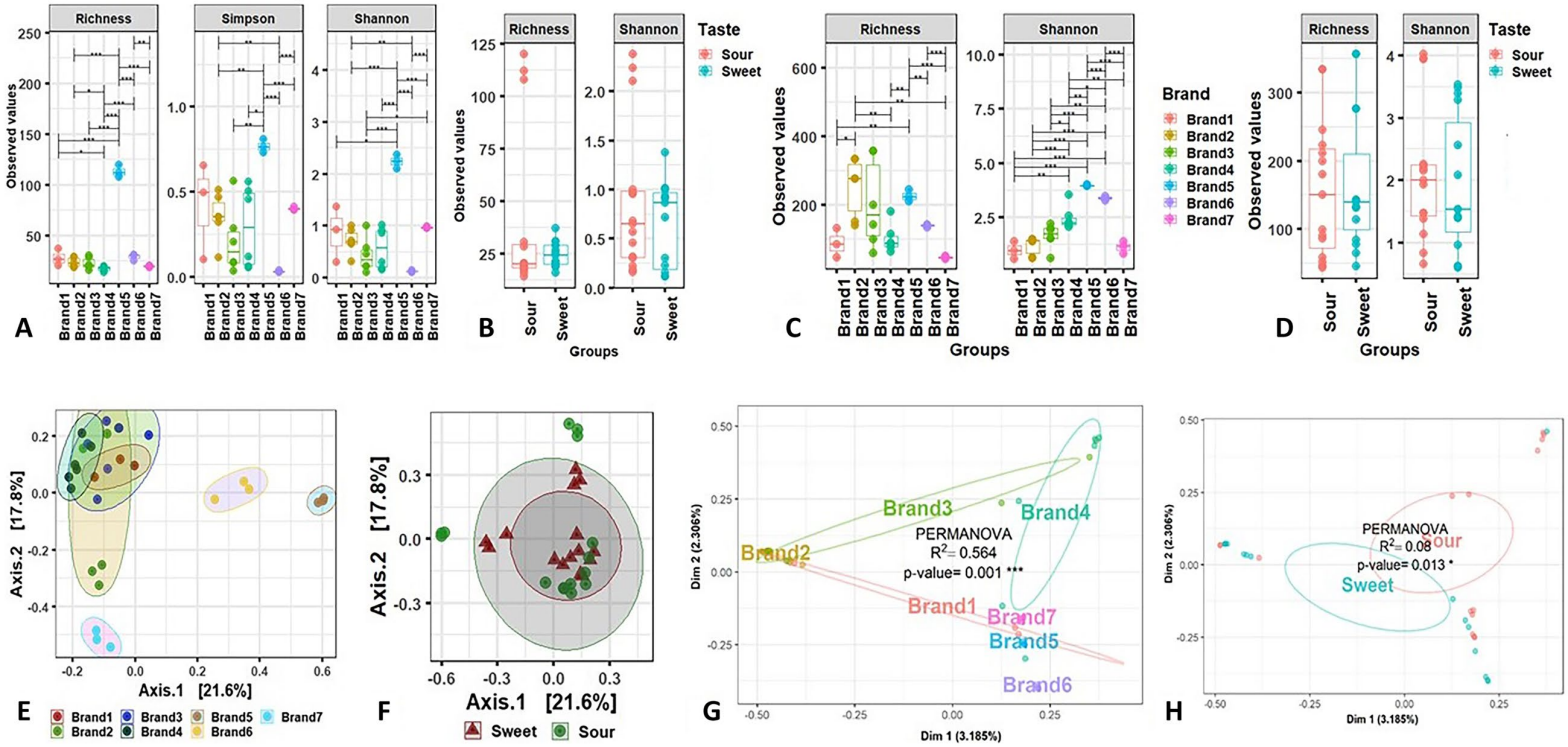
Differences in microbiome diversity and community structure in seven different brands (Brand 1–7) and two different tastes (sweet and sour) of yogurt samples. (**A**–**D**) Alpha diversity was measured through the observed species richness, Simpson and Shannon estimated diversity indices. (**A**,**B**) *α*-diversity of bacterial communities and (**C**,**D**) *α*-diversity of fungal fraction of yogurt microbiomes according to different brands (**A**,**C**) and tastes (**B**,**D**). (**E**–**H**) Beta-diversity was estimated through PCoA and NMDS plots. (**E**, **F**) Principal coordinate analysis (PCoA) to measure the *β*-diversity in bacterial component of the yogurt microbiomes segregated yogurt samples according to (**E**) seven different brands and (**F**) two different tastes (sweet and sour); (**G**,**H**) Beta ordination (NMDS) plots representing the fungal component of the microbiomes separated yogurt samples according to (**G**) different brands and (**H**) two different tastes (sweet and sour) categories. Statistical analysis using Kruskal–Wallis tests showed significant microbial diversity variation (*P* < 0.01 = *, *P* < 0.001 = ***). The figures were generated using phyloseq (version 1.34.0)^52^ and Vegan (version 2.5-7)^53^ packages of R^52^.

The alpha–beta diversity of microbiomes was analyzed to observe the differences in microbial composition and diversity in yogurt. Figure 2 shows the alpha–beta diversity of bacterial and fungal communities in yogurt samples of different brands and tastes types (sweet and sour). Both alpha (within sample) and beta (between sample) diversities in yogurt samples of seven different brands (Brand 1–7) and two different tastes (sweet and sour) were estimated using different diversity indices (Fig. 2). Significant differences (*P* < 0.001; Kruskal–Wallis test) were found in *α-*diversity (observed richness, Shannon and Simpson estimated) in bacterial components of the microbiomes across the samples of both sweet and sour yogurts from cow and buffalo, and different brands (Fig. 2A,B). This diversity difference was evidenced significantly (*P* < 0.001; Kruskal–Wallis test) by higher taxonomic resolution in sour yogurt especially in Brand 5 (Fig. 2A). Though the observed species and Shannon estimated diversity of the fungal fraction of the yogurt microbiomes varied (*P* < 0.001; Kruskal–Wallis test) among different brands, it was less diverse between the taste types (sour and sweet) of yogurt (Fig. 2C,D). Moreover, the *α-*diversity of the fungal component of the yogurt microbiomes was found more diverse in sour yogurt compared to sweet yogurt samples (Fig. 2D).

The principal coordinate analysis (PCoA) plots representing *β*-diversity showed significant differences (*P* = 0.001, Kruskal–Wallis test) in bacteriome composition among different brands (Fig. 2E). Significant differences (*P* = 0.001, Kruskal–Wallis test) were also observed in bacterial diversity between sweet and sour yogurt sample groups (Fig. 2F), representing the prevalence of different sets of bacteria in each sample category. Similarly, NMDS estimated beta-ordination plots depicting the diversity of the fungal fraction of the yogurt microbiomes showed significant variations too in different brands (*P*_permanova_ = 0.001) and tastes (*P*_permanova_ = 0.013) (Fig. 2G,H). For instance, Brand 3 maintained the highest beta-dispersion in relation to Brand 6, Brand 5, Brand 2, and Brand 7, respectively (Table S1). According to taxonomic composition and differential taxonomic analysis, the sequence of the brands was: Brand 3 > Brand 2 > Brand 4 > Brand 1 > Brand 7 > Brand 5 > Brand 6. Least significant clustering differences were detected between Brand 1 and 6, and Brand 1 and 2 (Fig. 2G, Table S1).

### Multivariate analysis and downstream bioinformatics

During data analyses, the detected OTUs were assigned into 11 phyla and 76 genera for bacteria, and 5 phyla and 70 genera for fungi. Among the bacterial phyla, 10 were detected in sweet yogurt of cow milk, 10 and 9 were in sour yogurt samples of cow and buffalo milk, respectively (Table S2). Firmicutes was the most abundant phylum with a relative abundance of 92.89% followed by Proteobacteria (7.03%) (Fig. S1A). By comparing the relative abundances of these predominant phyla, Firmicutes was found to be the most abundant phylum in both sweet (99.84%) and sour (86.36%) yogurt samples of cow milk. Conversely, Proteobacteria was the most abundant phylum in sour yogurt (61.46%) of buffalo milk followed by Firmicutes (38.12%) (Fig. S1A, Data S1). In addition, a total of 30 orders of bacteria were identified across these sample groups, of which 24, 25 and 21 orders were detected in sweet yogurt of cow, sour yogurt of cow and sour yogurt of buffalo, respectively (Table S2). Among the bacterial orders, *Lactobacillales* was found as the predominating order in sweet yogurt of cow (99.48%) and sour yogurt of cow (86.20%), while the sour yogurt of buffalo milk had higher abundance of *Enterobacteriales* (52.82%), *Lactobacillales* (37.48%) and *Aeromonadales* (5.52%) (Data S1).

**Figure 3:**
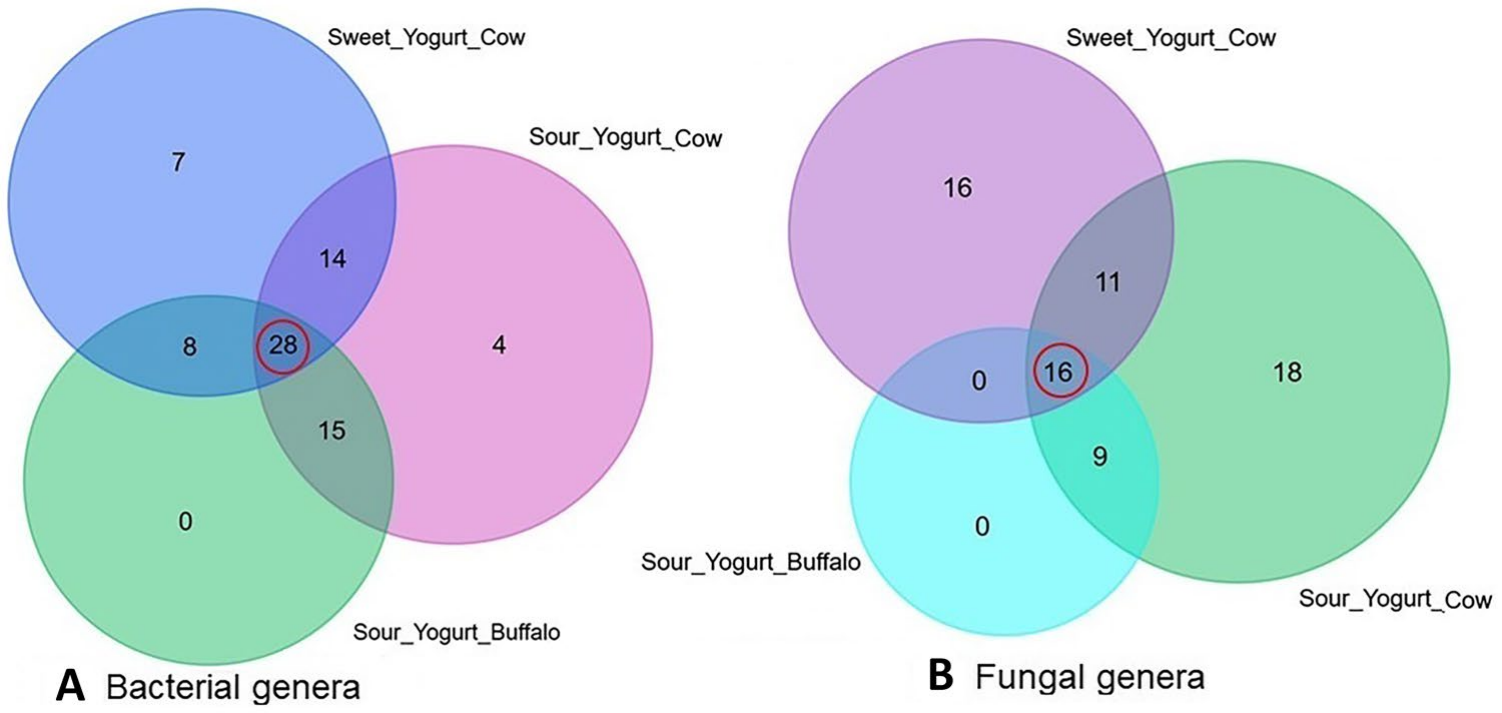
Taxonomic composition of microbiomes in two different tastes (sweet and sour), and milk sources (cow and buffalo) of yogurt samples. (**A**) Venn diagram showing unique and shared bacterial genera in sweet (cow) and sour (cow and buffalo) yogurt. Out of 76 detected genera, only 28 (36.84%) genera (highlighted in red circle) were found to be shared in sweet yogurt cow, sour yogurt cow and sour yogurt buffalo metagenomes. (**B**) Venn diagram comparison of unique and shared fungal genera identified in the study metagenomes. Of the detected fungal genera (n = 70), only 16 (22.86%) genera (highlighted in red circle) were found to be shared in the metagenomes of sweet yogurt cow, sour yogurt cow and sour yogurt buffalo samples. More information on the taxonomic composition and relative abundances is available in Data S1. Venn diagrams were generated through an online tool used for Bioinformatics & Evolutionary Genomics (http://bioinformatics.psb.ugent.be/webtools/Venn/).

The taxonomic classification and comparison of microbiomes at genus-level in yogurt samples of different tastes and milk sources are illustrated in Fig. 3. In this study, the yogurt samples collectively harbored 76 bacterial genera (Fig. 3A) and of them, *Streptococcus* (50.82%), *Lactobacillus* (39.92%), *Enterobacter* (4.85%), *Lactococcus* (2.84%) and *Aeromonas* (0.65%) were the top abundant genera (Data S1). In addition, notable differences were demonstrated in the diversity and composition of the identified bacterial genera both in sweet and sour yogurt samples of different brands. Among the detected bacterial genera, 36.84% genera were found to be shared in sweet yogurt of cow, sour yogurt of cow and sour yogurt of buffalo metagenomes (Table S2). The sweet and sour yogurt of cow had a sole association of 7 (9.2%) and 4 (5.26%) genera, respectively (Fig. 3A, Data S1). Likewise, 70 fungal genera were detected in the study samples, of which 22.86% genera were shared in the metagenomes of sweet yogurt cow, sour yogurt cow and sour yogurt buffalo (Fig. 3B; Table S2). Of the detected fungal genera in both sweet and sour yogurt samples, *Kluyveromyces* (65.75%), *Trichosporon* (8.21%), *Clavispora* (7.19%), *Candida* (6.71%), *Iodophanus* (2.22%), *Apiotrichum* (1.94%), and *Issatchenkia* (1.35%) were the most abundant genera (Data S1). The sweet and sour yogurt samples of cow had a sole association of 16 (22.86%) and 18 (25.71%) fungal genera. However, the sour yogurt samples of buffalo were observed to have no unique bacterial and fungal genera among the study metagenomes (Fig. 3B, Data S1).

**Figure 4:**
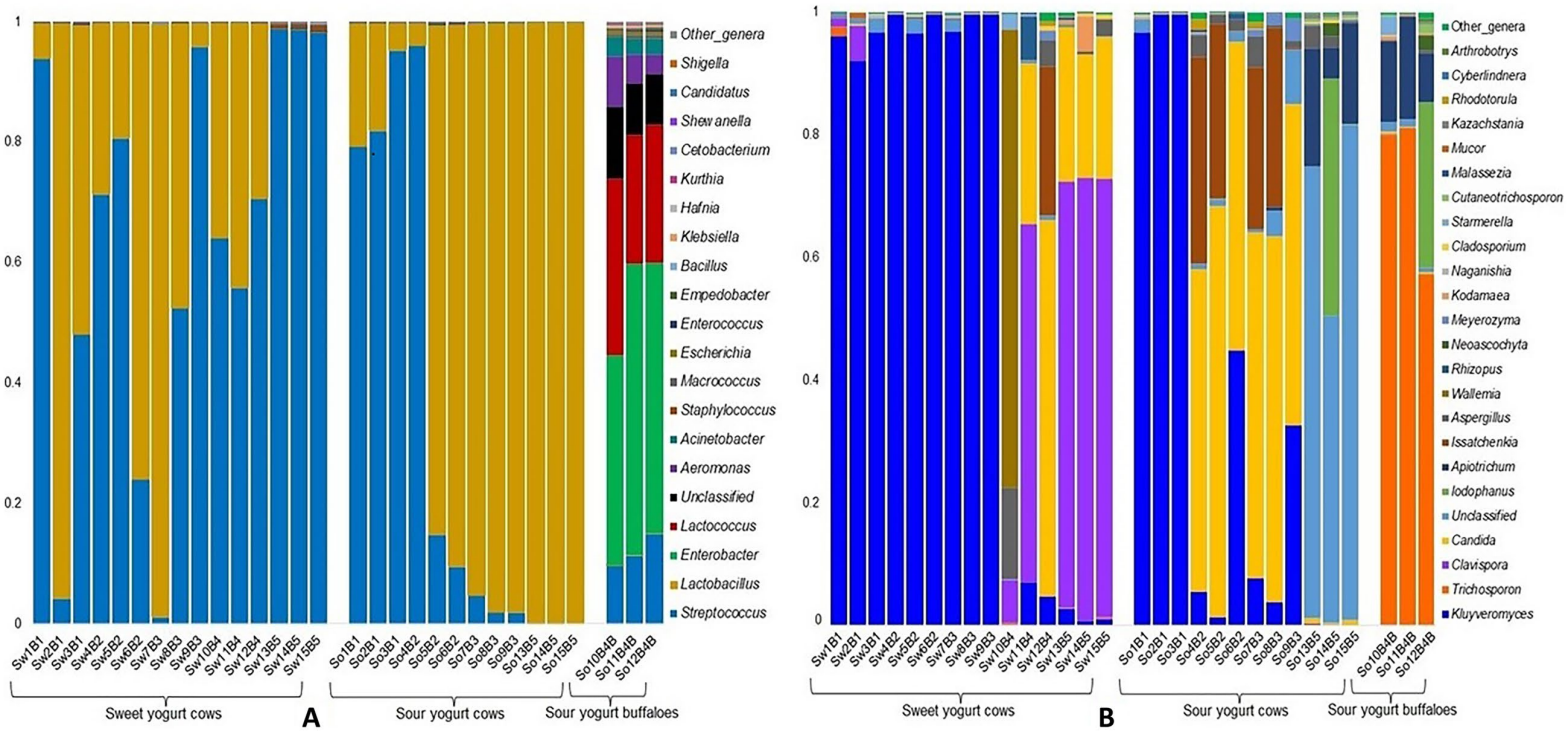
The genus level taxonomic profile of bacteria and fungi in sweet yogurt of cow, sour yogurt of both cow and buffalo samples. The relative abundance of (**A**) 20 most abundant bacterial and (**B**) 25 most abundant fungal genera are sorted from bottom to top by their deceasing proportion with the remaining genera keeping as ‘other genera’. Each stacked bar plot represents the abundance of bacterial and fungal genera in each sample of the corresponding category. Notable differences in bacterial and fungal populations are those where the taxon is abundant in sweet yogurt samples, and effectively undetected in the sour yogurt samples. The distribution and relative abundance of the bacterial and fungal genera in the study metagenomes are also available in Data S1. The bar plots are generated using Microsoft Excel program.

The taxonomic classification of bacteria and fungi was also analyzed at genus level in yogurt of various brands, tastes and milk source varieties (Fig. 4). Among the bacterial genera, *Streptococcus* (71.49%) and *Lactobacillus* (27.93%) had higher relative abundances in cow originated sweet yogurt samples (Fig. 4A; Data S1). On the other hand, sour yogurt samples of cow had higher relative abundances of *Lactobacillus* (50.34%), *Streptococcus* (30.32%), *Enterobacter* (9.28%), *Lactococcus* (5.41%) and *Aeromonas* (1.22%). Simultaneously, *Streptococcus* (42.43%), *Macrococcus* (24.76%), *Enterobacter* (11.88%), *Empedobacter* (9.76%), *Aeromonas* (5.54%) and *Enterococcus* (2.91%) were detected as the top abundant genera in sour yogurt of buffalo. The rest of the genera had relatively lower abundances (< 1.0%) in these metagenomes. Further investigation was performed to observe whether genus level relative abundances of the fungi differed between sweet and sour yogurt of different hosts (cow and buffalo) and brands (Fig. 4B). *Ascomycota* represented more than 98.0% of the total reads at genus level, except for Brand 5 and 6 that consisted of 59.8% of *Basidiomycota* (Fig. S1B). The sweet yogurt metagenome of cow origin had higher relative abundance of *Kluyveromyces* (78.45%), *Clavispora* (12.23%), and *Candida* (5.37%) while *Kluyveromyces* (47.62%), *Trichosporon* (19.74%), *Candida* (8.63%), *Iodophanus* (5.37%), *Apiotrichum* (4.70%), and *Issatchenkia* (2.71%) were the most abundant fungal genera in the sour yogurt metagenome of cow samples (Fig. 4B). Conversely, the sour yogurt sample of buffalo showed higher relative abundances of *Trichosporon* (70.14%), *Iodophanus* (12.07%), *Apiotrichum* (11.94%), and *Neoascochyta* (1.08%), and rest of the fungal genera detected in both sweet and sour yogurt metagenomes had lower relative abundances (< 1.0%) (Fig. 4B, Data S1).

**Figure 5:**
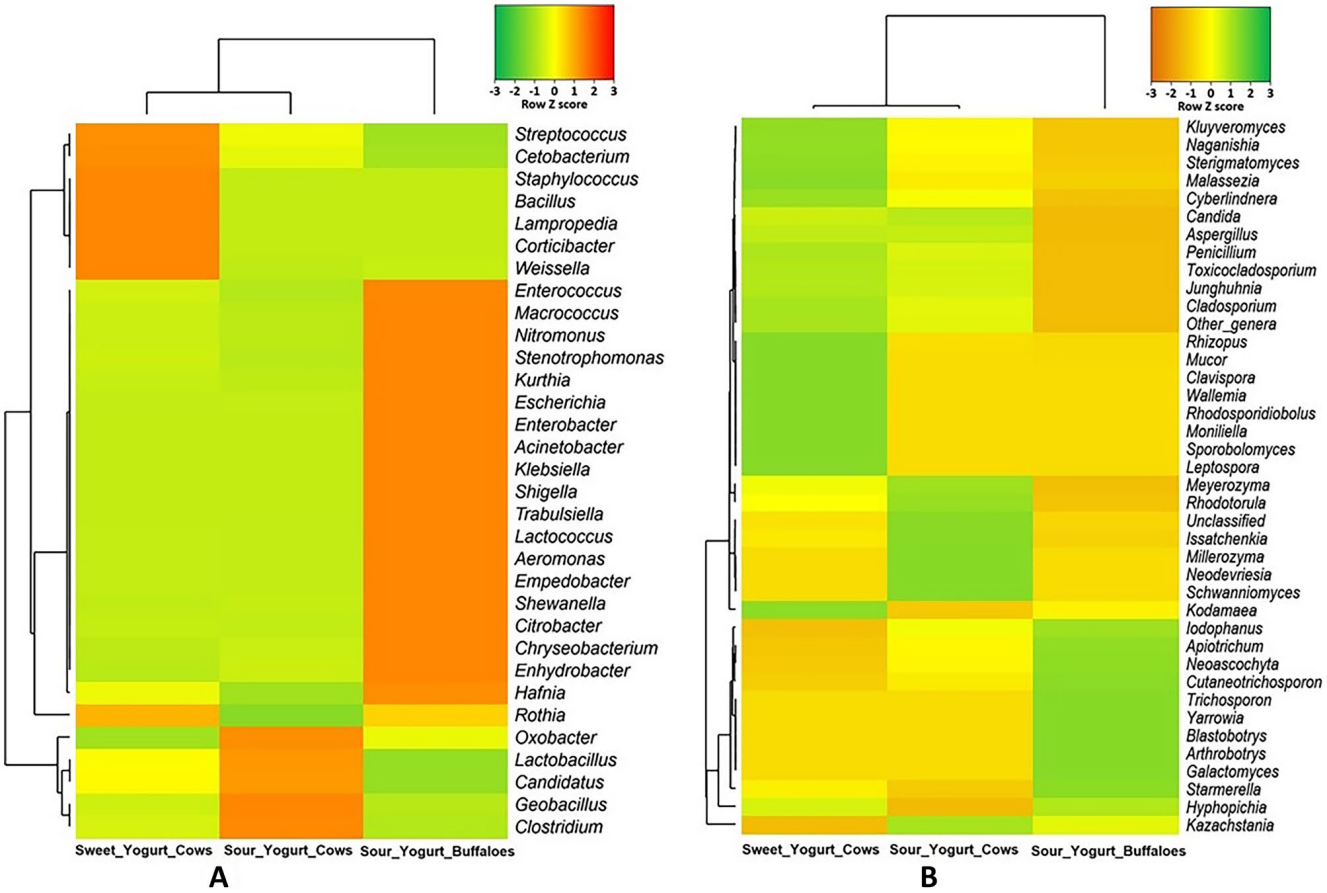
Genus level taxonomic differentiation of bacteria and fungi in sweet yogurt (from cow milk) and sour yogurt of cow and buffalo milk. Heatmap showing 40 top abundant bacterial genera across the sample categories. The color bars (column Z score) at the top represent the relative abundance of each (**A**) bacterial genus and (**B**) fungal genus in the corresponding group. Noteworthy differences in bacterial and fungal populations are those where the genus is abundant in either sweet or sour yogurt samples, and effectively not detected in other metagenomes. The color codes indicate the presence and completeness of each genus in the corresponding sample group, expressed as a value between − 3 (lowest abundance) and 3 (highest abundance). The red color indicates the more abundant patterns, while green cells account for less abundant putative genes in that particular metagenome. The Heatmap is built through a stand-alone software tool; FunRich (http://www.funrich.org/).

The heatmap representation (Fig. 5) of the yogurt microbiomes shows distinct separation among the detected bacterial genera into two major clusters according to their host origin i. e., cow and buffalo rather than tastes of the yogurt. The heatmap of relative abundance of bacteria and fungi at genus level across the tastes and source varieties of yogurt is also presented in Fig. 5. The cow originated bacterial genera of the yogurt microbiomes were further sub-clustered according to tastes (sweet and sour) (Fig. 5A, Data S1). This clustering was consistent with the bar plot (Fig. 4A) representation of bacteria since *Streptococcus* and *Lactobacillus* were the top abundant bacterial genera in cow originated sweet yogurt samples (Fig. 5A). Likewise, *Lactobacillus*, *Streptococcus*, *Enterobacter*, *Lactococcus* and *Aeromonas* were the predominating genera in sour yogurt samples of cow origin, and *Macrococcus*, *Enterobacter*, *Empedobacter*, *Aeromonas*, *Nitromonous*, *Stenotrophomonous*, *Kurthia Escherichia, Acinetobacter, Klebsiella, Shigella* and *Enterococcus* were predominantly abundant in buffalo originated sour yogurt (Fig. 5A). In addition, similar to bacterial genera, the detected fungal genera showed two distinct clusters in the heatmap representation (Fig. 5B) according to their host origin (i. e. cow and buffalo) rather than tastes of the yogurt. The cow originated fungal genera of the yogurt microbiomes were further sub-clustered according to tastes (sweet and sour) (Fig. 5B). The taxonomic distinction and clustering of fungal genera (Fig. 5B) according to hosts and tastes also corroborated with the results of bar plot (Fig. 4B) representation of fungal taxa. Differential abundance of bacteria and fungi in yogurt samples of different brands and tastes is listed in Table 3. Differential abundance analysis showed that *Aeromonas*, *Enterobacter*, *Lactococcus*, and *Acinetobacter* had significantly (Bonferroni corrected *P*_BC_ = 0.033–0.044, Bonferroni test) higher number of reads assigned for these genera in Brand 5 compared to other Brands (Table 3A). In addition, significantly (*P*_BC_ = 0.043, 0.024) higher number of reads assigned for *Streptococcus* and *Lactobacillus* were found in Brand 6 and 7, respectively. Significantly (*P*_BC_ = 0.028) greater number of *Streptococcus* associated reads (n = 4494.5) were observed in Brand 1 and this might be linked to the sweetness of yogurt brands (Table 3B).

**Table 3:** Differential abundance of bacteria and fungi in Bangladeshi yogurt based on brands and tastes.

Abundant genera	*P*_BC_-value	Differential abundance of microbes in yogurt						
		Brand 1	Brand 2	Brand 3	Brand 4	Brand 5	Brand 6	Brand 7
(A) Yogurt brands								
**Bacteria**								
*Lactobacillus*	0.024	3595	1971.2	3860	4704	6.3	3.3	**7049.7**
*Aeromonas*	0.033	0.0	2.3	2.0	0.8	**382.7**	3.3	0.0
*Streptococcus*	0.043	3440.3	5071.2	3170.8	2339.8	845.3	**6956**	0.8
*Lactococcus*	0.043	0.7	0.3	1.3	0.7	**1693.3**	7.7	0.0
*Enterobacter*	0.044	0.7	1.2	2.3	1.2	**3026.3**	4.3	0.0
**Fungi**								
*Apiotrichum*	0.033	0.0	0.0	0.0	7.4	552.7	0.0	**945.3**

Abundant genera	*P*_BC_-value	Differential abundance of microbes in yogurt						
		Sweet				Sour		
(B) Yogurt tastes								
**Bacteria**								
*Streptococcus*	0.028	**4494.5**				1986.7		
**Fungi**								
*Apiotrichum*	0.011	0.1				**302.5**		
*Clavispora*	0.047	**792.3**				0.2		

The microbial genera with more than 1% read abundance are presented only. Differential abundances of bacteria and fungi were analyzed by Kruskal–Wallis test and shown as mean values. Boldface values indicate most abundant microbial genera. The Bonferroni corrected *P*-value (*P*_BC_) < 0.05 is considered significant. Differential abundance is expressed in number (n). Kruskal–Wallis test followed by Bonferroni correction was done for analysis.

A differential abundance analysis of the fungal genera showed that higher number of reads (n = 945.3) mapped to *Apiotrichum* in Brand 7 (Table 3A). Similarly, higher abundance of reads assigned for *Clavispora* in sweet brands and *Apiotrichum* in sour brands might reflect the differences in the taste of commercial yogurt (Table 3B). To investigate the relationships between relative abundance of microbial genera and presences of minerals in both sweet and sour yogurt, we used non-parametric Kruskal–Wallis test which revealed significant differences (*P* < 0.05) between the given conditions. The higher level of Na, K, Ca and Mg in the Sour Brand 4 whereas Cu in the Sour Brand 2 was observed in this research. Both of these sour Brands (Brand 2 and 4) were mostly dominated by a single genus of bacteria (*Lactobacillus*; > 97.0%) and fungus (*Kluyveromyces*; 86.23%) (Data S1). By contrast, Fe rich Sweet Brand 6 was mainly dominated by *Streptococcus* (89.54%) and *Clavispora* (71.0%). The Zn enriched Sweet Brand 6 and Sour Brand 7 were mostly dominated by both of these bacterial (*Lactobacillus* and *Streptococcus*), and fungal (*Clavispora*) genera (Data S1).

## Discussion

The production of dairy products such as yogurt using cow and buffalo milk or mixtures of milk from both hosts could be a feasible strategy to promote and expand the dairy industry in Bangladesh. Moreover, profiling the yogurt types that are currently being manufactured in terms of nutritional value and microbiome can also highlight the scopes of improvement in the products, including quality and shelf-life. This study was, therefore, conducted to explore the nutritional quality assessment, level of mineral constituents or contamination and to explore the hub of microbial consortia in Bangladeshi yogurt of different brands (Brand 1–Brand 7) and taste types (sweet and sour). The biochemical analyses showed that the study samples had a high range of pH (5.28–6.33) which revealed a discordance with the previous report on yogurt pH value (4.6–5.06)^26^. The sour yogurt had comparatively lower pH than the sweet yogurt samples. The control of pH and acidity are undoubtedly important parameters in yogurt processing due to their functional contribution in curd coagulation, ripening, and shelf life. In sour yogurt, the decrease of pH could be associated with the fermentation of lactose to lactic acid by LAB^27^. Differences in pH values among the samples of two different taste types and seven commercial brands indicate likely influence of the rate of acidification during bacterial fermentation. The pH values of milk during the high heat treatment significantly influence many properties of the protein particles and their behavior in yogurt fermentation^28^. Moreover, improper incubation time and temperature in addition with regional and source differences might be linked with the high range of pH of the tested yogurt samples.

During this study, the average fat content of yogurt samples ranged from 0.25 to 2.52% (*w/w*) with no significant differences among the yogurt brands and tastes. Three samples showed individual fat percentages above 3.25, nevertheless, their mean difference did not show statistical significance. According to USDA and FDA statements, yogurt is labeled as non-fat, low-fat and regular if it contains less than 0.5%, 0.5–2.0% and at least 3.25% of fat, respectively^9,29^. The nutritive value of yogurt prepared from cow and buffalo milk with respect to MC, TS, SNF and ash ranged between 60–88%, 12–39%, 12–38%, and 0.8–1.25%, respectively. Majority of samples had an average MC below the normal range while having mean TS, SNF and ash that exceeded the average nutritive value^27^. Usually, the sour yogurt samples had higher MC than the sweet samples. Conversely, the mean TS and SNF content remained higher in sweet yogurt samples compared to the sour ones. However, an abnormal increase in the value of TS and SNF (e.g., in Sweet Brand 4) substantiates the removal of cream and addition of adulterants. This study did not reveal any significant difference in fat contents of the yogurt samples of both sweet and sour tastes and seven commercial brands.

The mineral contents of the yogurt samples of the current study showed distinct variations as evidenced by the higher value for Na content in sweet yogurt samples. Among different minerals, the lowest amount of Na was found in Sour Brand 4 and Sour Brand 3. The sour yogurt samples possessed a higher mean content of K, Ca and Mg. Variation for the nutritional value of minerals (Na, K, Ca and Mg) and trace elements (Fe and Zn) for low-fat and fat-free yogurt were found in the range of 700–770 mg/kg for Na, 2341–2552 mg/kg for K, 1829–1988 mg/kg for Ca, 170–188 mg/kg for Mg, 0.82–0.88 mg/kg for Fe and 5–10 mg/kg for Zn^29^. The imbalance in mineral content might be due to the adulteration of yogurt with unusual compounds. However, the mineral contents detected in the present study in most of the samples confirmed the role of yogurt as a source of essential nutrients in comparison with raw milk^27^.

Yogurt is a good source of trace elements such as Fe, Cu and Zn which are essential for human and animal metabolism and growth^9,29^. In this work, significant variations in the trace minerals content of the yogurt samples were observed and the mean content of Fe was always found to be higher in sweet yogurt samples (except for one Sour Brand 7). The highest accumulation of Zn was recorded in Sour Brand 7 while the least concentration of Zn was detected in Sweet Brand 4 yogurt. Likewise, the Cu content also varied among different brands of yogurt of two different tastes. The dietary reference intakes according to the World Health Organization (WHO) for Zn and Cu are 8–11 and 0.7–0.9 mg/kg, respectively^30^. Only two brands, Sweet Brand 6 and Sour Brand 7 were in the range of permissive value for Zn, while all others were below the range. The accumulation level of Cu in all samples crossed the reference value, while showing conformity among themselves.

In this study, 76 bacterial genera were detected which were mostly represented by increased phylum-level signature of *Firmicutes* (> 92.89%) and *Proteobacteria* (> 7%). *Streptococcus* and *Lactobacillus* (> 50% relative abundances) were detected as the most dominant LAB genera in all brands of sweet and sour yogurt. Strains of these two bacteria are generally known as the traditional starter bacteria which have been used for manufacturing yogurt since ancient times^8^. Moreover, some species of LAB are known to have beneficial impacts on the human gut, hence are being used as probiotics^3,4^. Microbial composition and diversity differ from raw to pasteurized milk, and between curd, whey, cheese and yogurt. The raw milk microbiota is influenced by microbes present in the teat canal, the surface of teat skin, hygiene practices, animal handlers, healthy and disease condition of the mammary glands, and the indigenous microbiota of equipment and storage containers^13–15^. Remarkably, the sour yogurt samples showed higher taxonomic abundances of *Lactobacillus*, *Streptococcus*, *Macrococcus*, *Enterobacter*, *Empedobacter*, *Lactococcus* and *Aeromonas* genera. This taste specific microbiomes discrimination was more evident in the sour yogurt samples of buffalo since most of the genera had higher relative abundances in buffalo yogurt metagenome. There is growing consensus that the microbiota is an ecosystem working together to keep humans healthy, with no specifically defined structure. The origin of the milk would also seem to influence the levels of diversity therein, with cow milk appearing to be more diverse than that from buffalo, goat and sheep^13,31^. However, until now, no substantial information is available regarding the breed specific association of microbiomes in yogurt samples of different tastes.

A notable link was observed between *Streptococcus* and sweetness of yogurt (*P*_BC_ = 0.028) in terms of taste of the yogurt showing accordance with recent findings on enzymatic modification of starter bacteria to derive sweetness enhanced yogurt^32^. This might be due to the lower incubation temperature (< 40 °C), shorter incubation period and higher pH along with the addition of sugar that favor the growth of *Streptococcus* over *Lactobacillus*^5^. Pathogenic bacterial genera reported in previous studies including *Pseudomonas*, *Micrococcus*^18^, *Enterococcus*, *Leuconostoc*, *Pediococccus*^19^ were detected at very low levels in this study (Data S1, not shown in Fig.). However, OTU reads assigned for *Aeromonas*, *Enterobacter*, *Lactococcus*, and *Acinetobacter* were found to be higher in Brand 5 while in other brands their relative abundances remained quite insignificant. *Aeromonas* genus exists in soil and aquatic environments, and is responsible for gastrointestinal infection in humans. It is also found to be associated with food-borne diseases like traveler’s diarrhea and water-borne disease outbreaks^33^. It is notable that bacteria comprising *Enterobacter* genus are fecal coliforms associated with bovine intestinal infection that emerge as contaminants in yogurt^34^. Species of the genus *Macrococcus* are naturally widespread commensals primarily isolated from animal skin and dairy products but becoming increasingly recognized as veterinary pathogens^14,15^, and thus could have dramatic consequences for public health. Though *Acinetobacter*, gram negative rods are present in the normal flora of human skin, they evolve as opportunistic pathogens and cause disease in immunocompromised patients via food or water^14,35^. *Lactococcus* along with *Acinetobacter* are reported to produce off-flavor, rancidity and ropiness in dairy products during storage^34^. Therefore, the presence of the above mentioned spoilage bacteria elucidates the quality of the yogurt samples analyzed in this work.

The present study marks an additional step towards identifying the significant co-occurrence of fungi with bacterial population in yogurt samples. In comparison to bacteria, the relative abundance and diversity of fungi remained slightly lower. Currently, there is no extensive evidence supporting the role of fungi in the fermentation of dairy products like yogurt. Significant differences were found in the composition and diversity of the fungal taxa across the sweet and sour yogurt metagenomes. The *Kluyveromyces*, a genus of *Ascomycetous* yeasts was found as the mostly abundant fungal genus in the metagenomes of both sweet and sour yogurt of cow origin while the sour yogurt of buffalo milk had the highest abundance of *Trichosporon*, a genus of Basidiomycota fungi. Fungal genera predominantly identified in these metagenomes were *Clavispora*, *Candida*, *Iodophanus*, *Apiotrichum*, and *Issatchenkia*. Fungal contamination is a frequent scenario in dairy industries indicating unhygienic practices during manufacturing of these products. Among the previously reported fungal genera- *Kodamaea*, *Clavispora*, *Candida*, and *Tricosporon*^18^, the first one was not detected in this research. In addition, OUT reads assigned to detected fungal genera of *Kluyveromyces*, *Trichosporon*, *Candida*, *Clavispora*, and *Apiotrichum* in this study were not reported earlier in Bangladeshi yogurt samples. The ability of the different species of the *Kluyveromyces* genus to metabolize milk constituents (lactose, proteins and fat) makes them very important in cheese and yogurt ripening and fermented milk products as they contribute to maturation and aroma formation^36^.

However, despite the importance of fungi in the dairy products, commercial yeast starters unlike LAB are not in routine use, and fungal flora developing in the yogurt and other dairy products appears as a result of spontaneous contamination, as they are able to grow in low pH, low storage temperature, low water activity, elevated salt concentration, and can even multiply during storage of product. Fungal spoilage generally occurs during final packaging of the finished product with the addition of ingredients and contributes to food-borne diseases in humans^5,36^.

Minerals are associated with bacterial physiological processes reshaping the gut microbiota’s structure and composition^37^. Fermented dairy products like yogurt are rich in many minerals with a higher bioavailability. The contribution of LAB with particular emphasis to *Streptococcus* and *Lactobacillus* seems to play an important role in the absorption of these minerals (Na, Ca, K, Mg, Cu and Zn), inhibition of other pathogenic microbiota, and in the stimulation of intestinal secretion for digestion of yogurt. Furthermore, fermentation with LAB to produce yogurt results in an acidic environment that can enhance the bioavailability of these minerals. The lower pH of the gut maintains Ca and Mg in their ionic forms, facilitating their enhanced absorption. The pH also facilitates the ligand binding affinity of zinc for transportation across the intestinal wall, resulting in increased absorption of Zn^37,38^. However, unlike *Saccharomyces*, the role of *Clavispora* with higher mineral contents, and their role in the bioavailability in yogurt need to be determined in vivo.

However, this study will serve as a benchmark for further work on the yogurt microbiome structure and quality manufactured by different companies. Future studies should focus on the contribution of the microbiota in the quality of yogurt using a larger scale of samples, and how yogurt and human gut microbiome interact to provide health benefits.

## Conclusion

To our knowledge, this was the first study to present a comparative analysis of the microbial flora in different yogurt types and brands available in Bangladesh. A higher mean pH value, along with elevated fat, moisture, TS and SNF contents (%, w/w) were found for sweet yogurt samples while sour yogurt samples had higher amounts of ash and minerals (Na, K, Ca, Mg, Zn and Cu). An acute dominance of bacterial genera *Lactobacillus* and *Streptococcus* were found in 6 out of 7 brands of yogurt of both taste types. A higher bacterial diversity and profusion of opportunistic pathogens including *Aeromonas*, *Enterobacter*, *Macrococcus* and *Lactococcus* were found in sour buffalo yogurt samples. *Kluyveromyces*, an *Ascomycetous* yeast genus, along with *Trichosporon*, a genus of *Basidiomycota* fungi was also detected in the samples in abundance indicating fungal contamination. The fungal diversity was more significant in sour buffalo yogurt. Although the results obtained from this study are substantial, it should be taken into account that the study was conducted with only a few samples, and was solely based on targeted metagenomics (e.g., 16S rRNA or ITS) which was unable to detect viruses and perform functional profiling. Large scale studies including more samples from all the regions and a range of state of the art analytical techniques are further required to observe and enumerate the spectrum of yogurt microbial flora.

## Methods

### Sampling

Thirty (n = 30) yogurt samples of cow (n = 27) and buffalo (n = 3) milk belonging to seven renowned brands (Brand 1–Brand 7) and two different taste types (sweet; n = 15 and sour; n = 15) were collected from different regions of Bangladesh (Fig. S2, Data S1, Table S1). For this study, seven sampling sites in Bangladesh, i.e. Rangpur, Bogura, Manikgonj, Dhaka, Munshiganj, Chattogram and Cox’s Bazar were selected (Fig. S2) based on the consumers demand of yogurt and its popularity in these selected areas. Aseptic condition was maintained during the collection and transport of products to the laboratory. The samples were randomly collected from the local confectionery shops, placed immediately in a cooling box containing refrigerants (at 4 °C) and transported (within 12 h) to the laboratory. Yogurt samples were stored at − 80 °C until further processing for subsequent experiments.

### Nutritional properties of yogurt

The nutritional properties of yogurt samples in terms of biochemical parameters (pH, fat, moisture content, total solid, solid-non-fat, and ash) and mineral contents were determined according to the standard methods^39,40^. All the biochemical analyses were carried out in triplicate and mean values ± standard deviations (SD) were recorded throughout the article.

#### Determination of biochemical parameters

The pH values of yogurt samples were determined at room temperature using a pH meter (Hanna Instruments, USA). The pH measurement was conducted three times for each sample and the average values were recorded. Fat content of yogurt sample was estimated according to the AOAC (Association of Official Analytical Chemists)^39^ by treating the sample with concentrated ammonia solution, then with ethanol, diethyl ether and petroleum ether respectively. The whole process was repeated three times to extract the whole fat content. The solvent was evaporated and the resulting fat sediment was oven-dried (at 100 °C for 2 h) to calculate the percentage (%, *w/w*) of fat from dry-weight.

The moisture content (MC) of a food product is defined as the mass of water present in a known mass of sample. The MC of yogurt was measured following the method described by AOAC^39^. Briefly, the initial weight of the yogurt sample was taken at a constant basis and then oven-dried at 105 °C for 3 h. After over-drying, the sample was immediately placed in a desiccator and the dry (final) weight was again taken. The MC was calculated by subtracting the dry weight from the initial weight of the sample and was expressed in percentage (%, *w/w*). The amount of total solid (TS) of yogurt was determined by gravimetric method as outlined in AOAC^39^ and was expressed in percentage (%). Solid-non-fat (SNF) is the value of leftovers after fat content is removed from dairy products. It was calculated by subtracting the estimated fat content (%) from TS (%). The ash content was estimated by incinerating the sample in a muffle furnace at 550 °C for 24 h according to the method of AOAC^39^ and was expressed in % (*w/w*).

#### Determination of mineral contents

After oven-drying and dry-ashing, the inorganic residues left in the crucible were digested with HNO_3_ and diluted up to 100 mL of volume in order to measure the mineral contents of yogurt samples. The diluted samples were then introduced to the Flame Atomic Absorption Spectrophotometer (iCE-3000 FASS, Thermo Fisher Scientific, USA) for the determination of minerals including Na, K, Ca, Mg, Fe, Zn and Cu. The wavelengths used to determine the contents of these minerals were 589.0, 766.5, 422.7, 285.2, 248.3, 213.9, and 324.8 nm, respectively. Standard solutions were prepared at four different concentrations of 0.25, 0.50, 1.0 and 2.0 ppm for all of these elements except Ca for which the concentrations were of 0.5, 1.0, 2.0 and 4.0 ppm^40^.

### Genomic DNA extraction and amplicon sequencing

The genomic DNA from different yogurt samples was extracted using a commercial microbiome DNA purification kit (Thermo Fisher Scientific, USA) following the manufacturer’s instructions. The amplification of bacterial DNA was achieved by targeting the V3–V4 region of 16S rRNA gene with 30 µL final volume containing 15 µL of 2 × master mix (BioLabs, USA), 3 µL of template DNA, 1.5 µL of each V3–V4 forward and reverse primers, 341F (5′-CCTACGGGNGGCWGCAG-3′) and 806R (5′-GACTACHVGGGTATCTAATCC-3′), respectively^41^ and the remaining 9 µL of DEPC treated ddH_2_O. A 25 cycle of PCR amplification including initial denaturation at 95 °C for 3 min, denaturation at 95 °C for 30 s, primer annealing at 55 °C for 30 s and elongation at 72 °C for 30 s was performed for bacterial DNA with the final extension of 5 min at 72 °C in a thermal cycler (Analytik Jena, Germany).

To amplify fungal DNA, the universal fungal primers ITS1-1F-F (5ʹ-CTT GGT CAT TTA GAG GAA GTA A-3ʹ)/ITS1-1F-R (5ʹ-GCT GCG TTC TTC ATC GAT GC-3ʹ) spanning the ITS1 region of rRNA gene were utilized^42^. PCR mixture for the amplification of fungal DNA was the same as the one used for bacteria. For fungal DNA, a thirty-five cycles of PCR amplification was run with the temperature profile of initial denaturation at 94 °C for 3 min, denaturation at 94 °C for 45 s, annealing at 57 °C for 1 min, elongation at 72 °C for 1.5 min and final extension of 10 min at 72 °C. After electrophoresis, the PCR amplicons were visualized in 1.5% agarose gel prepared in 1 × TAE buffer. The microbiomes of yogurt were assessed by HTS based on 16S and ITS genes. Agencourt Ampure XP beads (Beckman Coulter, Brea, USA) were used for PCR products purification, and the Nextera XT index kit (Illumina, San Diego, USA) for paired-end library preparation according to Illumina standard protocol (Part# 15,044,223 Rev. B). Paired-end (2 × 300 bp reads) sequencing of the prepared library pools was performed using MiSeq high throughput kit (v3 kit, 600 cycles) with an Illumina MiSeq platform (Illumina, USA)^43^.

### Data processing and downstream bioinformatics

FastQC pipeline was used to examine the primary quality of Illumina sequences of microbiomes^44^. Poor sequencing reads and adapter sequences were trimmed or removed via BBDuk (with options k = 21, mink = 6, ktrim = r, ftm = 5, qtrim = rl, trimq = 20, minlen = 30, overwrite = true)^14^. MeFiT (merging and filtering tool) was employed to efficiently merge overlapping paired-end sequence reads generated from the Illumina MiSeq with default parameters^45^. Filtering of merged reads, removal of chimeras, de novo assembly of reads into operational taxonomic units (OTUs) at 97% similarity level were performed using micca (microbial community analysis) OTU software pipeline (v1.7.0)^46^. Taxonomic classification of the representative bacterial OTUs was performed using micca classification against SILVA 1.32 release^47^, while fungal OTUs were classified against UNITE database^48^. PASTA algorithm^49^ and FastTree (v2.1.8)^50^ GTR + CAT model were used for multiple sequence alignment (MSA) and phylogenetic tree construction.

R software (v 4.1.1) was used for the downstream analysis including alpha–beta diversity, microbial composition and statistical comparison^51^. To estimate the within sample diversity (*α*-diversity), we calculated the observed OTUs, Shannon and Simpson diversity indices in microbiomeSeq (http://www.github.com/umerijaz/microbiomeSeq) and visualized using phyloseq R package (v1.34.0)^52^. To visualize differences in microbial diversity across the sample groups, a non-metric multidimensional scaling (NMDS) based on Bray–Curtis dissimilarity of weighted UniFrac metric, and permutational multivariate analysis of variance (PERMANOVA) was performed in Vegan^53^, microbiomeSeq and phyloseq (v1.34.0)^52^ package of R^52^. Differentially abundant bacteria and fungi at genus level were identified using non-parametric Kruskal–Wallis test followed by Bonferroni correction to exclude 5% false discovery rate (FDR) at 0.05 level of significance in QIIME (v1.9.1)^54^.

### Statistical analysis

All the experiments were carried out in triplicate and the data were expressed in average values ± SD. The statistical analyses were performed using the R software (version 4.1.1). Statistical significance in mean differences of various biochemical parameters of yogurt samples was examined using one-way analysis of variance (ANOVA). Post-Hoc test was performed for pair-wise comparison among different brand varieties and within themselves. The results were evaluated statistically with the probability (*P*)-value. At 95% confidence interval (CI), *P* < 0.05 was considered statistically significant. The non-parametric Kruskal–Wallis rank sum test was used to evaluate differences in the relative percent abundance of microbial taxa in different yogurt groups. Independent t-test was used to calculate the differences in microbial diversity between cow and buffalo yogurt.

### Supplementary Information

Supplementary Information 1.

Supplementary Information 2.

## Data Availability

All data analyzed during the present work are included in this article and its supplementary information. The raw sequence data in fastq files are currently available in National Centre for Biotechnology Information (NCBI) under the BioProject accession number PRJNA733702.
