# Author Correction: Insights into the nutritional properties and microbiome diversity in sweet and sour yogurt manufactured in Bangladesh

**DOI:** 10.1038/s41598-023-44099-2

**Published:** 2023-10-13

**Authors:** S. M. Rafiqul Islam, Afsana Yeasmin Tanzina, Md Javed Foysal, M. Nazmul Hoque, Meheadi Hasan Rumi, A. M. A. M. Zonaed Siddiki, Alfred Chin‑Yen Tay, M. Jakir Hossain, Muhammad Abu Bakar, Mohammad Mostafa, Adnan Mannan

**Affiliations:** 1 Department of Genetic Engineering and Biotechnology, Faculty of Biological Sciences, University of Chittagong, Chattogram, 4331 Bangladesh; 2 School of Molecular and Life Sciences, Curtin University, Bentley, WA 6102 Australia; 3 Department of Genetic Engineering and Biotechnology, Shahjalal University of Science and Technology, Sylhet, 3114 Bangladesh; 4 Department of Gynecology, Obstetrics and Reproductive Health, Bangabandhu Sheikh Mujibur Rahman Agricultural University, Gazipur, 1706 Bangladesh; 5 Department of Pathology and Parasitology, Chattogram Veterinary and Animal Sciences University, Chattogram, 4225 Bangladesh; 6 Helicobacter Research Laboratory, The Marshall Centre, University of Western Australia, Perth, WA 6009 Australia; 7 Forest Chemistry Division, Bangladesh Forest Research Institute, Chattogram, 4211 Bangladesh; 8 Bangladesh Council of Scientific and Industrial Research (BCSIR) Laboratories, Chattogram, 4220 Bangladesh

Correction to: *Scientific Reports* 10.1038/s41598-021-01852-9, published online 22 November 2021

The Supplementary Information 1 file published with this Article contained an error where there was a mistake in the sample name/identification number in the Supplementary File 1. The original Supplementary Information 1 file is provided below.

This error has now been corrected in the Supplementary Information 1 file that accompanies the original Article.

### Supplementary Information

Supplementary Information 1.

