# Supplementary material for: Insights into the nutritional properties and microbiome diversity in sweet and sour yogurt manufactured in Bangladesh
Source: Sci Rep. 2021 Nov 22;11:22667. doi: 10.1038/s41598-021-01852-9 (PMC8608820; doi:10.1038/s41598-021-01852-9)
Supplement: Supplementary file 2 — Supplementary Information 2. [file 41598_2021_1852_MOESM2_ESM.docx]

**Supplementary data**

**Insights into the nutritional properties and microbiome diversity in sweet and sour yogurt manufactured in Bangladesh**

S. M. Rafiqul Islam^1,# 🖂^ , Afsana Yeasmin Tanzina^1,#^, Md Javed Foysal^2,3^, M. Nazmul Hoque^4^, Meheadi Hasan Rumi^1^, AMAM Zonaed Siddiki^5^, Alfred Chin-Yen Tay^6^, S. M. Jakir Hossain^7^, Muhammad Abu Bakar^8^, Mohammad Mostafa^8^ & Adnan Mannan^1🖂^

^1^ Department of Genetic Engineering and Biotechnology, Faculty of Biological Sciences, University of Chittagong, Chattogram-4331, Bangladesh, E-mails: smrafiqulgeb@cu.ac.bd; [tanziageb@gmail.com](mailto:tanziageb@gmail.com); [rumicu0@gmail.com](mailto:rumicu0@gmail.com); adnan.mannan@cu.ac.bd

^2^ School of Molecular and Life Sciences, Curtin University, Bentley, WA 6102, Australia, E-mail: mdjaved.foysal@postgrad.curtin.edu.au

^3^ Department of Genetic Engineering and Biotechnology, Shahjalal University of Science and Technology, Sylhet-3114, Bangladesh

^4^ Department of Gynecology, Obstetrics and Reproductive Health, Bangabandhu Sheikh Mujibur Rahman Agricultural University, Gazipur-1706, Bangladesh,

E-mail: [nazmul90@bsmrau.edu.bd](mailto:nazmul90@bsmrau.edu.bd)

^5^ Department of Pathology and Parasitology, Chattogram Veterinary and Animal Sciences University, Chattogram-4225, Bangladesh, E-mail: [zsiddiki@gmail.com](mailto:zsiddiki@gmail.com)

^6^ Helicobacter Research Laboratory, The Marshall Centre, University of Western Australia, WA-6009, Australia; E-mail: alfred.tay@uwa.edu.au

^7^ Laboratory of Forest Chemistry, Bangladesh Forest Research Institute, Chattogram-4211, Bangladesh, E-mail: [smjakir080@yahoo.com](mailto:smjakir080@yahoo.com)

^8^ BCSIR Laboratories, Chattogram-4220, Bangladesh, E-mail: [abubakar-ctg@bcsir.gov.bd](mailto:abubakar-ctg@bcsir.gov.bd); drmostafabcsir@yahoo.com

^#^ These authors contributed equally: S. M. Rafiqul Islam and Afsana Yeasmin Tanzina.

^🖂^ Corresponding author: [smrafiqulgeb@cu.ac.bd](mailto:smrafiqulgeb@cu.ac.bd); [adnan.mannan@cu.ac.bd](mailto:adnan.mannan@cu.ac.bd)

**Supplementary tables**

**Table S1.** PERMANOVA of beta-dispersion for bacterial and fungal diversity in Bangladeshi yogurt of different brands and tastes.

| **Bacteria** | |  | **Fungi** | |
| --- | --- | --- | --- | --- |
| **Yogurt brands** | ***P*_permanova_ -value** |  | **Yogurt brands** | ***P*_permanova_ -value** |
| Brand 2, Brand 5 | 6e-06*** |  | Brand 3, Brand 6 | 1e-05*** |
| Brand 2, Brand 7 | 1e-05*** |  | Brand 3, Brand 5 | 3e-05*** |
| Brand 2, Brand 6 | 8e-05*** |  | Brand 2, Brand 3 | 3e-05*** |
| Brand 3, Brand 5 | 1e-03*** |  | Brand 3, Brand 7 | 1e-04*** |
| Brand 3, Brand 7 | 2e-03** |  | Brand 2, Brand 4 | 6e-03** |
| Brand 2, Brand 4 | 2e-03** |  | Brand 4, Brand 6 | 7e-03** |
| Brand 4, Brand 5 | 2e-03** |  | Brand 4, Brand 5 | 1e-02* |
| Brand 3, Brand 6 | 4e-03** |  | Brand 4, Brand 7 | 3e-02* |
| Brand 4, Brand 7 | 5e-03** |  | Brand 1, Brand 2 | 3e-02* |
| Brand 4, Brand 6 | 2e-02* |  | Brand 1, Brand 6 | 3e-02* |
|  |  |  |  |  |
| **Yogurt tastes** | ***P*_permanova_ -value** |  | **Yogurt tastes** | ***P*_permanova_ -value** |
| Sweet – Sour | 0.016 |  | Sweet – Sour | 0.013 |

Significance levels of *P*_permanova_ -values are depicted as *P* < 0.01 = *, *P* < 0.001 = **

and *P* < 0.0001 = ***.

**Table S2.** Taxonomic distribution of bacteria and fungi in yogurt of different tastes, source variety and brands.

| **Taxonomic rank** |  | **Taste and source variety of yogurt** | | |
| --- | --- | --- | --- | --- |
|  |  | **Sweet yogurt cow** | **Sour yogurt cow** | **Sour yogurt buffalo** |
| **Bacteria** |  |  |  |  |
| Phyla (n=11) |  | 10 | 10 | 9 |
| Order (n=30) |  | 24 | 25 | 21 |
| Genus (n=76) |  | 57 | 61 | 51 |
| **Fungi** |  |  |  |  |
| Phyla (n=5) |  | 5 | 4 | 3 |
| Order (n=28) |  | 23 | 24 | 14 |
| Genus (n=70) |  | 43 | 54 | 25 |

**Supplementary figures**


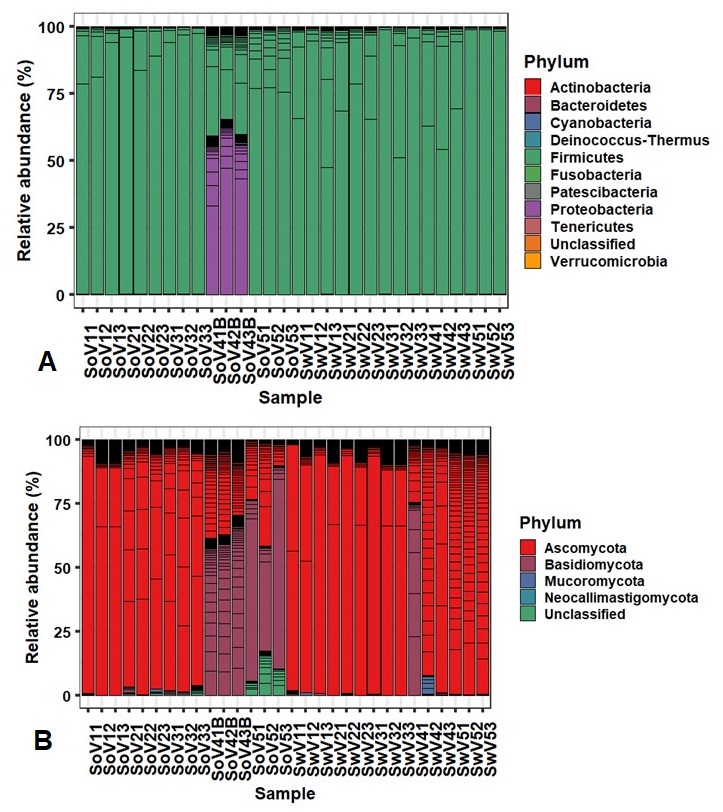


**Fig. S1.** Relative abundance of microbial communities at phyla level in yogurt of different brands and taste varieties: **(A)** bacteria and **(B)** fungi. Each stacked bar plot represents the abundance of microbial phylum in sample of the corresponding variety. Those who are not phylogenetically assigned to any phylum are left ‘unclassified’.


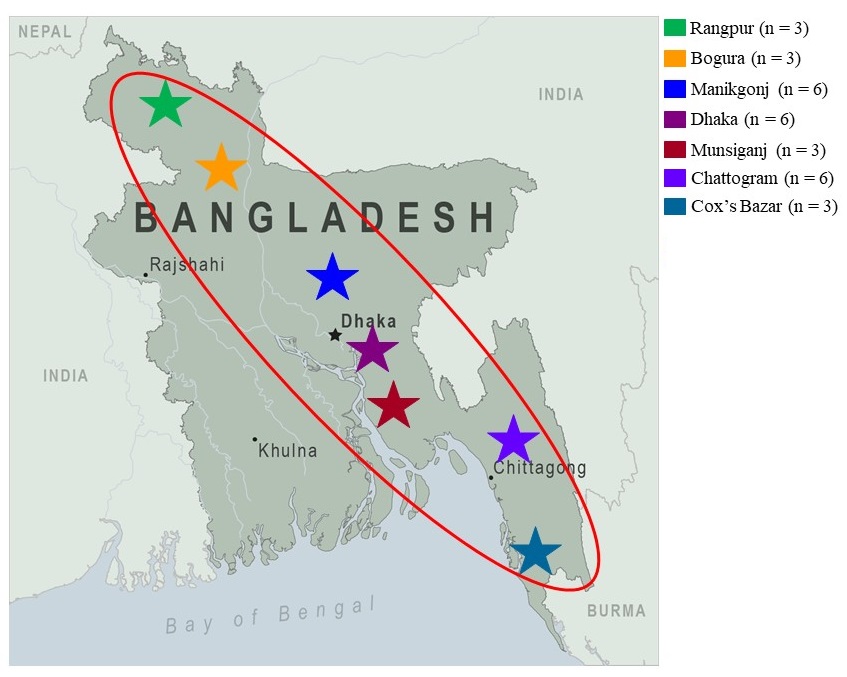


**Fig. S2.** Location of sampling sites in the seven distinct regions of Bangladesh. The red circle comprises the yogurt sampling sites represented in different color codes. The Figure is generated using Google Map i.e., districts map of Bangladesh (http://1.bp.blogspot.com/d5tHPRb1hz0/UNyc9ljbIvI/AAAAAAAAACM/eo8Pvw44Hk8/s1600/BangladeshDistrictsMap.JPG) following the Attribution Guidelines for Google Maps (<http://www.google.com/permissions/geoguidelines/attr-guide.html>) and Microsoft PowerPoint.
